# Relationship Between Attainment of Recommended Physical Activity Guidelines and Academic Achievement: Undergraduate Students in Egypt

**DOI:** 10.5539/gjhs.v6n5p274

**Published:** 2014-07-15

**Authors:** Walid El Ansari, Christiane Stock

**Affiliations:** 1 Faculty of Applied Sciences, University of Gloucestershire, Gloucester, United Kingdom; 2 Unit for Health Promotion Research, Institute of Public Health, University of Southern Denmark, Esbjerg, Denmark

**Keywords:** physical activity, academic achievement, university students

## Abstract

**Introduction:**

We assessed and compared by gender, students’ achievement of recommended guidelines of four PA forms, and the association between guideline achievement of each of the four PA forms and students’ academic performance.

**Methods:**

Data (2009-2010) comprised 3,271 students (11 faculties) at Assiut University, Egypt. A self-administered questionnaire measured: moderate PA (MPA), vigorous PA (VPA), moderate to vigorous PA (MVPA), muscle-strengthening PA; five socio-demographic variables (gender, age, year of study, father’s education, living arrangements during semester); self-rated health; and, academic performance. We compared the levels of four PA forms, socio-demographic variables, and academic performance by gender. Binary logistic regression examined the factors associated with achieving the guidelines of the four PA forms. Linear regression examined the association between frequency of four PA forms and level of academic performance.

**Results:**

Nearly equal proportions of males and females (37%, 36%) achieved the MPA guidelines. Significantly more males achieved the VPA, MVPA, and muscle strengthening PA guidelines. Father’s education was positively associated with achieving all four PA guidelines (with each increasing educational achievement of the father, student’s odds of achieving PA guidelines increased by 7-9%). Students living with their parents or room mates off campus were more likely to achieve the VPA and MVPA guidelines. Students who achieved VPA and MVPA guidelines were more likely to report better academic performance. For all PA forms (except MPA), increasing academic achievement was positively associated with increasing frequency of PA, but standardised Beta (0.05-0.07) suggested a modest correlation between academic achievement and PA frequency.

**Conclusion:**

The linear association between frequency of PA and academic achievement, and the finding that the proportions of students who achieved the recommended levels of several forms of PA were below half of the sample call for higher engagement of universities in fostering PA and active lifestyle among students.

## 1. Introduction

Health is a critical element for academic achievement (Tsouros, Dowding, Thompson, & Dooris, 1998) and exercise seems associated with improved academic outcomes. Despite this, 40%-50% of college students were physically inactive (Keating, Guan, Piñero, & Bridges, 2005).

For adolescents, many studies reported positive associations between physical activity (PA)/cardiovascular fitness and academic performance/attainment (Shin & So, 2012; Van Dusen, Kelder, Kohl, Ranjit, & Perry, 2011; Booth et al., 2014; Ardoy et al., 2014); whilst others found no support that greater PA improved achievement (LeBlanc et al., 2012). In terms of college students, the relationship between academic achievement and PA is in partial contrast to school children. Researchers found no direct association between PA and academic grade (Al-Kandari & Vidal, 2007), even when using both subjective and also objective academic achievement measures (El Ansari & Stock, 2010). However, others found a higher grade average in students meeting moderate to vigorous PA (MVPA), to advocate that those who adhere to public health lifestyle recommendations have modestly higher grade averages after adjusting for sociodemographic and negative health behaviors (Wald, Muennig, O’Connell, & Garber, 2014).

A range of factors is important for PA. These include socio-demographic variables (e.g. gender, age, year of study, father’s education, living arrangements during university terms) and self-rated health. Studies examined gender, income, employment status, and education levels, and found that the older students had lower exercise scores (Hui, 2002); and research of achievement of lifestyle recommendations (e.g. PA) and academic performance adjusted for sociodemographic and negative health variables (Wald et al., 2014).

As for gender, research findings seem to be inconsisent in terms of gender differences in the relationships between academic achievement and PA. For instance, for adolescents, there was no association between PA and academic performance in boys, but such association was present in girls (Martínez-Gómez et al., 2012). Others reported that among Swedish 9th-grade school students, in girls, academic achievement was associated with vigorous PA (not mediated by fitness), whereas in boys only fitness was associated with academic achievement (Kwak et al., 2009). A recent study of Korean adolescent students found that vigorous PA was positively correlated with academic performance in boys, and moderate PA was positively correlated with academic performance in both genders (So, 2012). In addition, recent reports observed that the association between academic achievement and PA was gender-dependent, and was subject to the PA intensity (Ayan Cancela Carral, & Montero, 2013). Genetic markers might reveal any dynamism from poor health to lower academic achievement, and any heterogeneity in their impacts across genders (Ding et al., 2009).

The literature reveals gaps. First, PA and academic attainment has been extensively examined in school children, but not university students (Al-Kandari & Vidal, 2007; El Ansari & Stock, 2010). In school settings, movement can promote children’s cognitive development (Reed et al., 2010), triggering the need to evaluate the fitness-academic performance connections (Van Dusen et al., 2011). For university students, similar studies are lacking and urgently required. Secondly, there is scarcity of Eastern Mediterranean Region (EMR) studies of students’ health promoting lifestyles, and their relationship with academic performance (Al-Kandari & Vidal, 2007). Such studies have been undertaken to a wider extent in the USA, Europe and Far East (El Ansari & Stock, 2010; Wald et al., 2014), with very few EMR studies. Virtually no research examined these issues across Egyptian university students. This study bridges these knowledge gaps, and these features attach high importance to the contributions of the current research.

The current study surveyed a representative sample of undergraduate students at Assiut University, Egypt (11 faculties) to assess and compare by gender, students’ attainment of guidelines of four PA forms: moderate PA (MPA), vigorous PA (VPA), MVPA, and muscle-strengthening PA (MSPA). We also examined the associations between academic achievement (academic performance compared with one’s peers) and attaining the guidelines of each of the four PA forms while considering five socio-demographic variables (gender, age, year of study, father’s education, living arrangements during university terms) and self-rated health. The specific objectives were to:

- describe and compare the levels of four forms of PA, socio-demographic variables, and academic achievement by gender;
- assess the variables associated with achieving the recommended guidelines of each of the four forms of PA of the sample; and
- assess whether the frequency of PA is associated with academic achievement

## 2. Materials and Methods

### 2.1 Sample and Data Collection

The university research ethics committee provided ethical approval. Self-administered questionnaires were distributed to students attending lectures of randomly selected courses. Participation was voluntary, anonymous and data were confidential. Data comprised 3,271 students [104 with gender not recorded, leaving 1,504 males (47.5%) and 1,663 females (52.5%)] at 11 faculties (Business, Engineering, Education, Arts, Social Work, Sciences, Physical Education, Computers and Information, Veterinary Medicine, Specific Education, and Agriculture). Student’s mean age was 18.9 years (SD 1.42). By completing the questionnaire, students agreed to participate. Data entry was undertaken by one individual to minimize data entry errors. Based on the number of returned questionnaires, the response rates were about ≈90%.

### 2.2 Health and Wellbeing Questionnaire

The questionnaire gathered general health data: self-reported socio-demographic information (gender, age, year of study, living arrangements during university terms, socioeconomic status); lifestyle behaviours (four PA forms - MPA, VPA, MVPA, and MSPA); and university related questions (academic achievement in comparison with peers). We also examined self-rated health (potentially associated with PA and with academic achievement). The questionnaire has been used and field-tested across many student populations (El Ansari & Stock, 2010; El Ansari, Stock, & Mikolajczyk, 2012;^14,11^; El Ansari, Oskrochi, & Stock, 2013; El Ansari, Dibba, Labeeb, & Stock, 2014; El Ansari, Khalil, Crone, & Stock, In Press).

*Sociodemographic variables*:

*Age*, *gender* and *year of study* at university were based on self-reports.

*Living arrangements during university terms*: “Where do you live during university/college term time?” (alone, outside university campus; with my parents; on university campus; other).

*Socioeconomic status* (SES): “What is the highest education level of your father?” (7 levels: “No formal education,” “Primary school,” “Secondary school,” “High school,” “Bachelor’s degree,” “Master’s degree,” and Ph.D. or equivalent”).

*PA variables*:

*VPA*: “On how many of the past 7 days did you participate in vigorous exercise for at least 20 minutes?” Participants answered with 0–7 days. We used a cut-off of ≥ 3 days/week in line with American Heart Association guidelines (Haskell et al., 2007).

*MPA*: “On how many of the past 7 days did you participate in moderate exercise for at least 30 minutes?” Participants answered with 0–7 days. We used a cut-off of ≥ 5 days/week (Haskell et al., 2007).

*MVPA*: computed by combining together moderate PA and vigorous PA. Students who achieved either moderate or vigorous PA at recommended level were set as achieving MVPA (Haskell et al., 2007).

*MSPA*: “On how many of the past 7 days did you do exercises to strengthen or tone your muscles, such as push-ups, sit-ups, or weight lifting?” Participants answered with 0–7 days. We used a cut-off of ≥ 2 days/week (Haskell et al., 2007).

*Educational and health variables*:

*Educational achievement*: “How do you rate your performance in comparison with your fellow students?” (five-point Likert scale, “much worse” to “much better”) (El Ansari & Stock, 2010).

*Self-rated general health*: “How would you rate your health in general?” (“poor”, “fair”, “good”, “very good”, “excellent”) (Potthoff et al., 1999).

### 2.3 Statistical Analysis

Analyses were performed using the statistical package SPSS 14.0 (p < 0.05). For the demographic and PA variables, frequencies were calculated separately for males and females. Gender comparisons were undertaken using chi-square (χ^2^) statistics for categorical variables. Binary logistic regression analyses examined factors associated with achieving recommended levels of four PA forms (MPA, VPA, MVPA, MSPA) as dependent variables while adjusting for gender and all other variables in the model. We included faculty as dummy variable to test for cluster sampling effects. Since faculty had no effect on the estimates, this variable was not included in the final models (to increase the models’ power). For the association between the frequency of the four forms of PA (per week) and level of academic achievement compared to peers (from much worse to much better), we performed linear regression analyses while adjusting the models for age, sex, father’s education and self-rated general health. Pearson correlation coefficients for all these variables entered into the model were < 0.2, suggesting that multicollinearity was not a substantial problem.

## 3. Results

### 3.1 Sociodemographic and Academic Characteristics of the Sample by Gender

Most students were ≤ 20 years old, with very few students ≥ 30 years old, highlighting the nature of study across universities in Egypt, where a substantial proportion of students are traditionally aged (i.e. ‘fresh’ from high school).

The majority of the sample was in their first or second academic year (Table 1). Males were significantly more represented in higher years of study, and also significantly more represented in the 21–29 years old age bracket than females. Significantly more females (64%) lived on campus during term time (26% males). Slightly more than a third of students reported that the father had Bachelor’s degree, with no differences between genders. A total of 31% of males (28% females) rated their own academic performance either somewhat or much better in comparison to peers, with no significant difference between genders.

**Table 1:** Sample characteristics and achievement of recommended PA guidelines for the whole sample and by gender

	Sample	Male	Female	P

	N=3271 (%)	N=1504 (%)	N=1663 (%)	

Socio-demographic and academic variables				
School year				< 0.001
Preparatory	22 (<)	10 (<)	12 (<)
1st Year	1058 (33)	547 (36)	505 (30)
2nd Year	818 (29)	326 (21)	590 (35)
3rd Year	862 (27)	424 (29)	421 (25)
4th Year	329 (10)	182 (12)	146 (9)
5th or 6th Year	28 (< 1)	207 (14)	149 (9)	
Age (Years)				< 0.001
≤ 20	2800 (86)	1164 (79)	1555 (95)	
21-29	470 (14)	315 (21)	85 (5)	
≥ 30	1 (< 1)	0 (0)	1 (< 1)	
Living arrangements (during semester)				< 0.001
Alone/with room mates*^a^*	522 (16)	437 (30)	62 (4)	
With parents*^a^*	1218 (38)	652 (44)	532 (33)	
On university campus	1456 (46)	385 (26)	1038 (64)	
Education of father				0.073
No formal education	543 (17)	285 (19)	255 (15)	
Primary school	369 (12)	174 (12)	193 (12)	
Secondary school	242 (8)	120 (8)	121 (7)	
High school	801 (25)	354 (24)	444 (27)	
Bachelor’s degree	1109 (35)	514 (34)	593 (36)	
Master’s degree/Ph.D.	99 (3)	51 (3)	46 (3)	
Rating of one’s own academic performance compared to peers				0.151
Much less	80 (3)	34 (2)	44 (3)	
Somewhat less	461 (14)	212 (14)	247 (15)	
About the same	1750 (54)	810 (53)	932 (56)	
Somewhat better	790 (25)	387 (26)	398 (24)	
Much better	133 (4)	75 (5)	58 (4)	

Health and physical activity variables				

Self-rated health				< 0.001
Poor	153 (5)	74 (5)	75 (5)	
Fair	961 (30)	382 (25)	570 (37)	
Good	1531 (47)	719 (47)	798 (47)	
Very good	433 (13)	238 (16)	191 (11)	
Excellent	164 (4)	112 (7)	51 (3)	

Achieve guidelines of moderate PA*^b^*	979 (36)	436 (37)	509 (36)	0.449
Achieve guidelines of vigorous PA*^b^*	778 (33)	471 (41)	304 (26)	< 0.001
Achieve guidelines of MVPA	1066 (48)	591 (54)	472 (42)	< 0.001
Strengthening exercises ≥ 2 times/week*^b^*	912 (42)	625 (56)	282 (27)	< 0.001

a outside university campus;

b as recommended by guidelines (Haskell et al., 2007).

### 3.2 Achieving Recommended Guidelines of Four Forms of PA by Gender

**Figure 1:**
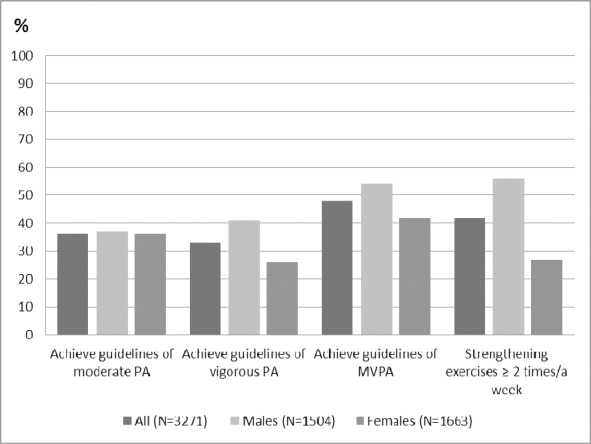
Achievement of recommended PA Guidelines for whole sample and by gender

About the same proportion of males and females (37%, 36%) achieved the recommended MPA level (Table 1). More males (41%) than females (26%) achieved the VPA, the MVPA (54% males, 42% females), and the MSPA guidelines (56% males, 27% females) (Figure 1).

### 3.3 Variables Associated With Achieving Recommended Guidelines of Four Forms of PA

When adjusted for gender and other variables, some variables were associated with achieving recommended guidelines of all four PA forms (Table 2). Father’s education was consistently positively associated with achieving all PA guidelines except with MPA, where with each increasing educational achievement of the father, the student’s odds of achieving recommended PA levels increased by 7–9%.

**Table 2:** Variables associated with achieving recommended guidelines of four forms of PA

	Moderate PA	Vigorous PA	Moderate/Vigorous PA	Muscle strengthening PA

	Exp(B) 95% C.I.	Exp(B) 95% C.I.	Exp(B) 95% C.I.	Exp(B) 95% C.I.
Living arrangements				
On university campus	1.00	1.00	1.00	1.00
Alone/with room mates^a^	1.08 (0.84-1.38)	**1.26 (0.88-1.56)**	**1.41 (1.06-1.86)**	0.89 (0.67-1.18)
With parents^a^	0.84 (0.65-1.08)	**1.23 (1.29-1.86)**	**1.58 (1.29-1.93)**	0.90 (0.73-1.13)

Gender				
Male	1.00	1.00	1.00	1.00
Female	1.05 (0.87-1.25)	**0.57 (0.46-0.69)**	**0.72 (0.57-0.85)**	**0.26 (0.21-0.32)**

Age				
< 20	1.00	1.00	1.00	1.00
20-23	0.88 (0.72-1.08)	1.01 (0.81-1.27)	0.90 (0.73-1.13)	**0.77 (0.61-0.96)**
> 23	0.76 (0.35-1.62)	**0.31 (0.11-0.94)**	**0.37 (0.16-0.92)**	0.65 (0.27-1.56)

Education of father				
≤ High school degree	1.00	1.00	1.00	1.00
University degree or PhD	1.03 (0.87-1.22)	**1.35 (1.15-1.66)**	**1.24 (1.06-1.51)**	1.19 (0.90-1.31)

Academic year				
1st or 2nd year	1.00	1.00	1.00	1.00
Higher academic year	**1.23 (1.02-1.47)**	**1.25 (1.01-1.53)**	**1.31 (1.08-1.60)**	1.17 (0.97-1.47)

Academic performance compared to peers				
Lower or equal	1.00	1.00	1.00	1.00
Better	1.10 (0.93-1.31)	**1.36 (1.17-1.72)**	**1.28 (1.12-1.63)**	1.17 (0.96-1.42)

Self-rated health				
Poor/fair/good	1.00	1.00	1.00	1.00
Very good/excellent	1.06 (0.87-1.31)	**1.38 (1.11-1.72)**	**1.37 (1.10-1.70)**	**1.34 (1.07-1.68)**

Based on logistic regression analysis, odds ratios controlled for all other variables shown in the table; bolded cells indicate statistical significance; C.I. = Confidence Interval.

Students living on campus had the least odds of achieving the recommended levels of two PA forms (VPA and MVPA). Participants were more likely to fulfil recommended VPA and MVPA if living with their parents (compared to those living on campus). Those who lived alone or with roommates outside campus were also more likely to achieve recommended VPA and MVPA levels (compared to those living on campus).

Females were significantly less likely to achieve the PA guidelines except for MPA. Older students were significantly less likely to achieve the recommended VPA, MVPA and MSPA. However with increasing year at university, students were more likely to achieve the recommended MPA, VPA and MVPA levels. Students with better academic achievement and students who rated their health better were significantly more likely to achieve the VPA and MVPA guidelines. Likewise students reporting very good/excellent self-rated health were more likely to achieve the VPA, MVPA and MSPA guidelines compared to those who poor/fair/good self-rated health.

Based on logistic regression analysis, odds ratios controlled for all other variables shown in the table; bolded cells indicate statistical significance; C.I. = Confidence Interval.

### 3.3 Association Between Frequency of PA and Academic Achievement

For all forms of PA (except MPA), increasing perceived academic achievement was positively associated with increasing frequency of PA (Table 3). This association was highest for VPA. The standardised Betas for all three associations were relatively small (0.05–0.07) indicating a modest correlation between academic achievement level and PA frequency.

**Table 3:** Association between academic performance and frequency of four forms of PA

	Moderate PA frequency (days per week)	Vigorous PA frequency (days per week)	Moderate/Vigorous PA frequency (days per week)	Muscle strengthening PA frequency (days per week)
	
Adjusted R^2^ of model	0.003	0.022	0.019	0.114
Academic performance compared to peers^a^	0.004 (p=0.828)	**0.071 (p=0.001)**	**0.045 (p=0.040)**	**0.053 (p=0.013)**

Standardised Beta coefficients based on linear regression analysis adjusted for age, sex, education of father and perceived (self-rated) health; bolded cells indicate statistical significance;

a five categories from much worse to much better.

## 4. Discussion

Health and well being are crucial for effective learning, and education and health outcomes are interdependent. To the best of our knowledge, no data exists from Egypt on such relationships.

As for objective one, 48% of the sample achieved the recommended MVPA guidelines (54% males, 42% females). These findings are favourable when compared with university students: in Egypt, where one third of the sample were physically inactive (Abolfotouh, Bassiouni, Mounir, & Fayyad, 2007); in the USA, where the prevalence of meeting public health MVPA recommendations was 41.9% (Wald et al., 2014). Similar research supports a generally low level of PA across these young adults (Muttappallymyalil et al., 2010).

About 33% of our sample achieved the VPA guidelines (41% males, 26% females), comparing nicely with an Egyptian study where 16% of participants (30% males, 5% females) achieved VPA guidelines (Abolfotouh et al., 2007). We also compared favorably with Libya, where 11.2% of students (22.1% males, 5.6% females) achieved the VPA guidelines (El Ansari et al., In Press). For MSPA, 42% of our sample achieved the guidelines of this form of PA, where more males (56%) achieved the guidelines than females (27%). These levels are comparable with the USA where 32.4% of students met the public health recommendations of strength training (Wald et al., 2014). However, in our sample, MSPA might have been underestimated. The PA subscale item asked: “On how many of the past 7 days did you do exercises to strengthen or tone your muscles, such as push-ups, sit-ups, or weight lifting?” Egypt is of predominantly Muslim faith, where it is customary for individuals to pray five times each day. This necessitates a succession of kneeling and standing over a period of time and can comprise a form of stretching exercises. In agreement, possibly our participants did not view praying as a form of exercise.

As for gender, our females were significantly less likely to achieve the recommended PA guidelines (except for MPA). Such gender differences agree with Libya (El Ansari et al., In Press), and with earlier research in Egypt (Abolfotouh et al., 2007). Generally, less females achieve the PA recommendations: in Mexico and Hong Kong male university students were physically more active/exercised more frequently (Ulla Díez & Pérez-Fortis, 2010; Lee & Yuen Loke, 2005). In the EMR, it is not common to see individuals jogging in the streets, and even much less so for females, due to prevalent cultural norms, or also influenced by religion for Muslim females living in predominantly Muslim societies. The consequence is that females might have fewer opportunities than males to engage in PA.

For objective two, father’s educational attainment (measure of socio-economic status, SES), was positively associated with VPA and MVPA. We are in support of Mexico, where students with a medium-high socio-economic level reported more activity (UUlla Díez & Pérez-Fortis, 2010). At the general population level, lack of exercise was more prevalent in the lower social classes (Wardle & Steptoe, 2003). Likewise, higher non-compliance in terms of adherence to PA recommendations for adolescents was associated with low socioeconomic status (Galán et al., 2013). Certainly, data from 32 countries indicated that in all but 7 countries, children with higher Family Affluence Scale level had significantly higher MVPA (Borraccino et al., 2009).

Nevertheless, our findings contrast with earlier research of university students in Egypt (Abolfotouh et al., 2007), where compared to high SES students, moderate and lower SES students were both less likely to exhibit lack of exercise. A point is that whilst we employed father’s education as an SES indicator, the Egyptian study (Abolfotouh et al., 2007) operationalized SES as a combination of several indicators (education and occupation of parents, family size, housing conditions and family income). While we only focused on the educational component of SES, the wealth component of a combined SES may be associated with lower levels of PA

As for the other variables (gender, year at university, age), the Spanish HBSC survey reported being female; being older; and not having a high level of academic achievement as factors associated with higher non-compliance to the recommendations for PA in adolescents (Galán et al., 2013). We are in agreement, as we found that females were significantly less likely to achieve the recommended PA guidelines (except for MPA where no gender differences were noted); older students were significantly less likely to achieve recommended levels of VPA, MVPA and MSPA; and those with academic achievement better compared to their peers were more likely to achieve the VPA and MVPA guidelines. Likewise, students reporting very good/excellent self-rated health were more likely to achieve the VPA, MVPA and MSPA guidelines compared to those with poor/fair/good self-rated health.

For objective three, we found that for all forms of PA (except MPA), increasing academic achievement was positively associated with increasing frequency of PA. Others have reported that when the MVPA guideline was met, student’s grade average was higher (Wald et al., 2014), highlighting the relative importance of PA. PA seems to also have ‘dual’ effects: habituated exercise fostered academic performance; and it also buffered the combined effects of stress, anxiety and depression on academic performance (Hashim, Freddy, & Rosmatunisah, 2012). Whereas we found increasing academic achievement was positively associated with increasing frequency of PA, this positive association was strongest for VPA. Nevertheless, the strengths of all three associations were relatively small (0.05-0.07), suggesting a weak correlation between level of academic achievement and frequency of PA i.e. PA makes a significant but modest contribution to better academic performance. Others found the association between academic achievement and PA to depend on PA intensity (Ayan et al., 2013).

The study has limitations. We examined 11 faculties at one university, generalizations should be cautious. Selection bias cannot be ruled out. We operationalized selected variables, but other variables could be associated with PA: e.g. biological (BMI, parental BMI), social parental (income), behavioural (TV/computer habits, sports club membership, time outdoors), physical environmental (neighbourhood features), psychological (self-esteem) and seasonal features. This study is cross-sectional; no causal relationships can be derived. Self-reporting estimated the PA levels and academic performance; objectively measured PA was not undertaken. There is need to attain a valid/less expensive instrument that can be used in large university-based studies to assess students’ PA behaviour.

## 5. Conclusion

This study suggested that PA with its documented positive effects on health and wellbeing has also the potential to contribute to a positive academic development among Egyptian students. The linear association between frequency of PA and academic achievement in combination with the finding that the proportions of students achieving recommended levels of several forms of PA were below 50% of the sample call for a higher engagement of universities in fostering PA among students. This could be undertaken through awareness raising campaigns and investments in sports facilities and other opportunities for PA. In addition, social or cultural barriers to PA should be assessed and reduced if present and fesaible. A special focus should be set in terms of engaging females more in PA.
